# Small fluctuations in cell wall thickness in pine and spruce xylem: Signal from cambium?

**DOI:** 10.1371/journal.pone.0233106

**Published:** 2020-05-21

**Authors:** Eugene A. Vaganov, Elena A. Babushkina, Liliana V. Belokopytova, Dina F. Zhirnova

**Affiliations:** 1 Siberian Federal University, Krasnoyarsk, Russia; 2 Sukachev Institute of Forest, Siberian Branch of the Russian Academy of Sciences, Krasnoyarsk, Russia; 3 Khakass Technical Institute, Siberian Federal University, Abakan, Russia; Chinese Academy of Sciences, CHINA

## Abstract

In the conifer tree rings, each tracheid goes through three phases of differentiation before becoming an element of the stem water-conducting structure: division, extension, and cell wall thickening. These phases are long-lasting and separated temporally, especially cell wall thickening. Despite the numerous lines of evidence that external conditions affect the rate of growth processes and the final anatomical dimensions during the respective phases of tracheid differentiation, the influence of the environment on anatomical dimensions during the cell division phase (cambial activity) has not yet been experimentally confirmed. In this communication, we provide indirect evidence of such an effect through observations of the small fluctuations in the latewood cell wall thickness of rapidly growing tree rings, which exhibit a high cell production rate (more than 0.4 cells per day on average). Such small fluctuations in the cell wall thickness cannot be driven by variations in external factors during the secondary wall deposition phase, since this phase overlaps for several tens of latewood cells in the rings of fast-growing trees due to its long duration.

## Introduction

The relative simplicity of the anatomical structure of conifer xylem, which consists of more than 90% the radial files of tracheids [1, 2, 3], has attracted the attention of not only wood anatomists, but also other scientists: ecophysiologists, biophysicists, biomathematicians, information technology specialists, technical engineers, etc. [4, 5, 6, 7, 8, 9, 10, 11]. In the sequence of tracheids in the radial file, each cell can be characterized by simple basic dimensions: the radial diameter (D) and cell wall thickness (CWT) [12, 13, 14]. Together with the total number of cells in a tree ring (N), these characteristics can also be integrated into other characteristics frequently used in dendroclimatology, e.g., tree-ring width and maximum wood density [15] It is well known that each tracheid, before becoming a functional element of the conifer xylem, passes through three stages of differentiation: 1) cell production by xylem mother cells in the cambial zone, 2) cell expansion, and 3) cell wall thickening, i.e., synthesis and lignification of the secondary cell wall [12, 16, 17, 18, 19]. A number of recent sophisticated works on seasonal growth kinetics made it possible to more thoroughly assess the dynamics of the cell number in the cambial zone, the cell expansion zone, and the maturation (cell wall thickening) zone, ending with apoptosis [18, 20, 21, 22]. Estimates of the duration of cell expansion are obtained for individual tracheids, generally decreasing from ~20 to ~10 days during the season; similar estimations of cell wall thickening indicate the opposite pattern of increasing duration from ~10 days in earlywood to >30 days in latewood [23, 24, 25]. Moreover, the duration of the respective growth process has a greater contribution than its rate to the final D, and the contribution of rate and duration to CWT is similar [7, 23, 26, 27]. The analysis of seasonal kinetics is important for identifying and understanding the external signal perception during xylem formation and its “recording” in the final tree-ring anatomical structure. In several of our works, it was clearly shown that morphometric parameters of tracheids perceive growth-limiting effects of climatic factors for short intervals during the growing season [28, 29, 30]. However, it is still unresolved which of the three phases of tracheid differentiation is the most sensitive to external influence [12, 31, 32, 33].

In this study, we considered this question based on tracheidograms (intraseasonal dynamics of cell morphometric parameters) of tree rings producing various numbers of cells per ring. We hypothesized that extremely wide tree rings as high-resolution images of cell parameters’ intra-seasonal variation can provide proxy assessment of the contribution of climatic conditions during corresponding and previous stages of tracheid differentiation to this variation even in absence of direct observations of its kinetics. Since cell production in tree ring (radial growth) generally decreases in colder conditions [34, 35] and is depressed at any environmental limit of the species growth [12, 36], we selected lower part of forest zone in South Siberian mountains (habitat with relatively warm and moderately dry conditions) as convenient testing ground to find trees with wide range of cell number per ring and significant climatic influence.

## Materials and methods

**Fig 1:**
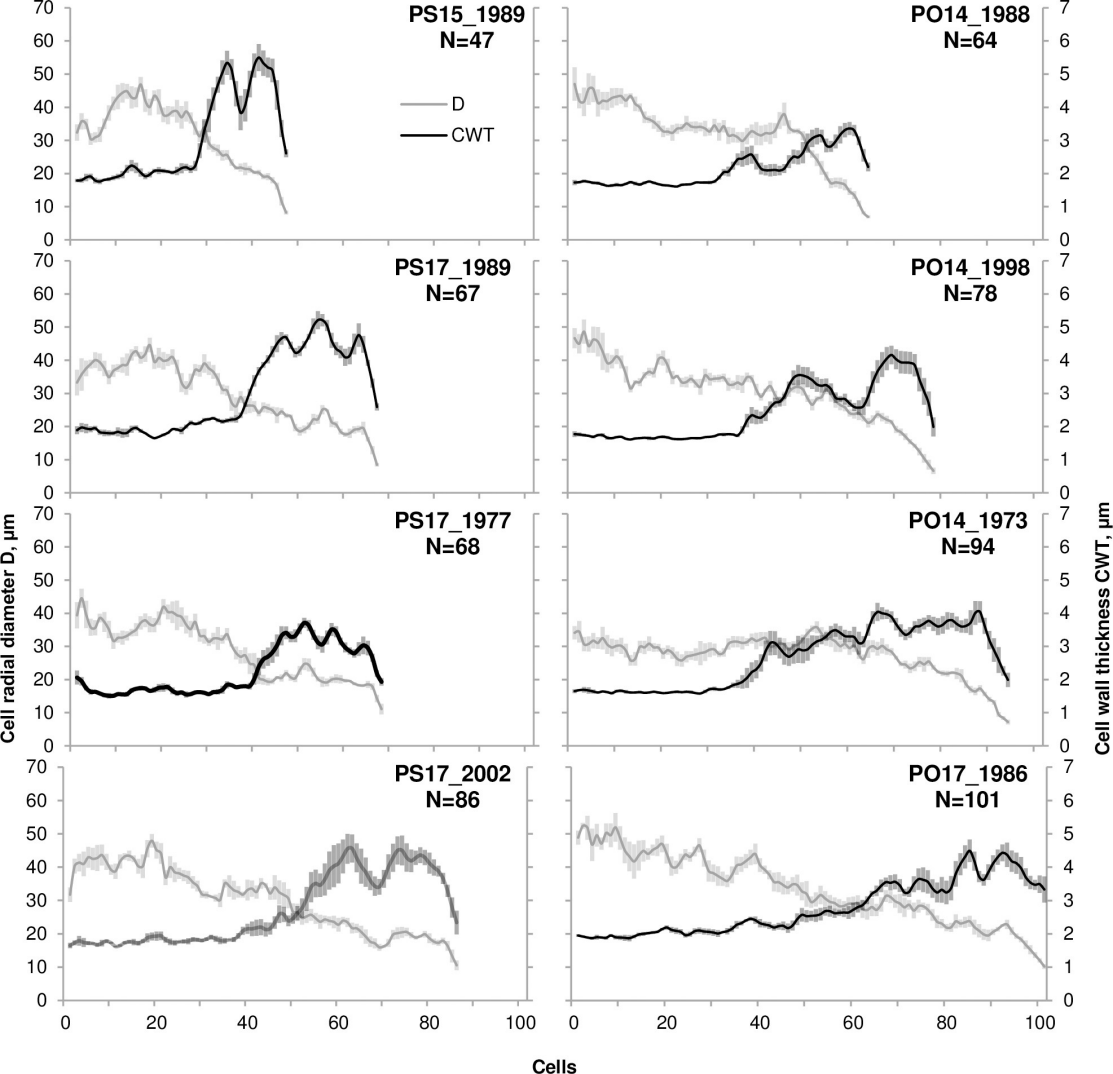
Examples of tracheidograms for wide rings. D, cell radial diameter (gray lines), CWT, wall thickness (black lines). Tree rings of *Pinus sylvestris* (individual trees PS15 and PS17) are presented in the left column of panels, tree rings of *Picea obovata* (individual trees PO14 and PO17) are presented in the right column of panels. In each column, panels are sorted with the cell number N increasing from top to bottom. Shaded error bars represent the *SE* range calculated from 5 measured radial files of tracheids.

The study was conducted in the foothills of the Borus Ridge, Western Sayan (South Siberia, Russia), in the lower part of the species altitudinal range in the region. The sampling site (52.83°N 91.45°E, 500–550 m a.s.l.) is located in the valley of the small Talovka River with 10–25° slopes facing south–north, in the “Shushensky Bor” National Park. The forest stand at the site is mixed: Scots pine (*Pinus sylvestris* L.), Siberian larch (*Larix sibirica* Ledeb.), Siberian spruce (*Picea obovata* Ledeb.), common aspen (*Populus tremula* L.), and silver birch (*Betula pendula* Roth.). For anatomical measurements, cores of 5 spruce trees (at the river bank and bottom of the northern slope) and 5 pine trees (at the bottom of the southern slope) were selected from larger number (~30 cores from 15–18 trees of each species) collected for dendrochronological purposes in 2015 by standard techniques [37]. Permission for sample collection was given by Tolmachev V.A., Director of the "Shushensky Bor" National Park. Involved in the study species are not endangered or protected. Adult dominant healthy trees were sampled, and cores selected for anatomical measurements were from trees of age >80 years (to exclude juvenile wood from consideration) and tree diameter at breast height 35–50 cm. N, D, and CWT were measured on the microphotographs of safranin-stained thin (<20 μm) cross-sections for five radial files in each ring over 50 years (1965–2014, a total of 250 rings for each species) with an accuracy of 0.01 μm, using Lineyka software [38]. This program manually or semi-automatically provides consequent measurements of double call wall and lumen along the selected path for the particular radial file of cells in the image of tree ring, and then transforms them in series of D and CWT. To allow generalization between 5 radial files with different N values, the tracheidograms of D and CWT were normalized (i.e., stretched or compressed [39]) to the average N in each ring. In the CWT tracheidograms, in addition to the general seasonal trend (stationary value in earlywood, gradual increase during transition to latewood, and decrease for the last tracheids), fluctuations with small amplitudes and lengths were observed in latewood. For each ring, the number of such fluctuations was counted. For distinction of small fluctuations, we used the *mean±SE* range of CWT over the same cell in 5 measured files, counting as the fluctuation deviations of CWT from the seasonal trend exceeding this range. Deviations of the same direction in several consequent cells were counted as one fluctuation. The observed fluctuations lasted on average 6–8 cells; in some rings, they were accompanied by synchronous fluctuations in D (Fig 1).

As the data sources on the seasonal kinetics of xylogenesis and the possible temperature thresholds, we used 3-weekly direct observations for both pine and spruce by micro-core sampling at the same site in 2019 (unpublished data) and daily temperature series from the Cheryomushki weather station (52.87°N 91.42°E, 330 m a.s.l., 5 km from site) smoothed by a 21-day moving average. We also used earlier 10-day observations of pine xylem phenology under relatively similar conditions (2013 and 2014, 53.65°N 91.58°E, 320 m a.s.l.; [10, 40]) to overcome low temporal resolution of local data, and compared these observations with the temperature data from the Minusinsk weather station (53.68°N 91.67°E, 260 m a.s.l., 9 km from site).

The climate of the study region is sharply continental [41]. At the Cheryomushki station, the average temperature of the cold season (T<0°C, November-March) is 5–11°C below zero, the average temperature of the warm season is +11–13°C, and the annual precipitation is 360–540 mm. To take into account the elevation of the sampling site, we adjusted the temperature series using the estimate of the temperature lapse rate of 0.65°C per 100 m [42, 43].

In previous studies we found significant climatic response in radial growth and wood anatomy of both species in the study area. Cell production and radial growth is limited mainly by soil water availability in May-June (positive correlations with precipitation and negative ones with temperature), latewood CWT has positive correlation with temperature in the end of summer [29, 44, 45].

## Results and discussion

### Assessment of seasonal kinetics

The comparison of the seasonal kinetics of conifer xylogenesis in the study region during several seasons ([10, 40], also unpublished data) with the corresponding temperature series showed that the threshold temperatures coinciding with the onset of cambial activity are approximately 8°C for both species (cf. range of threshold temperatures 5.6–8.5°C reported by Rossi et al. [46]). According to data from the Cheryomushki station (1951–2015), at the sampling site, this threshold usually occurs in the first half of May: *mean*±*SD* = 128±8 DOY (April 30 –May 16). On the other hand, cambial activity in all observations for the region ended at the end of July–beginning of August (210–220 DOY, 29 July– 8 August), probably due to the regulation of growth cessation by daylength [47, 48, 49]. Since the early onset of cambial activity is associated with a longer duration due to the gradual regulation of the growth process rates by morphogens [14, 20, 21, 50, 51, 52], the most likely duration of cell production in the study area can be estimated as 75–101 days. However, this estimation can be exceeded if spring is particularly early (cf. early onset of pine cambial activity on April 11 (101 DOY) and its duration of 110 days in 2014 at the other site [40]).

In fast-growing trees, a large number of cells can undergo cell wall thickening at the same time, especially in latewood. Simple estimates show that with the duration of cambial activity of 75–101 days and, for example, N = 100 and 50 cells, the average cell production rates would be 0.99–1.33 and 0.50–0.67 cells per day, respectively. From aforementioned studies, we may take 30 days as a modest estimate of the duration of the cell wall thickening for latewood tracheids in the study area. Then for our two examples of N, 30–40 and 15–20 cells have partially overlapping period of cell wall thickening. However, it is logical to expect increased N as a result of increased duration of cambial activity, making the lower boundary of these estimations more realistic. Indeed, direct observations of seasonal kinetics in the study region showed that the maximum number of cells in the zone of cell wall thickening occurred in August (i.e., for latewood), which accounted for 20–34% of the total cell production, i.e., 20–34 and 10–17 cells, respectively, for the given examples of N ([40] and unpublished data). This finding can also be partially explained by the fact that the cell production rate is not stationary during the season, and latewood cells are produced closer to the end of the cambial activity period, when this rate already decreases below its average value.

### Observations of the small CWT fluctuations in latewood

**Fig 2:**
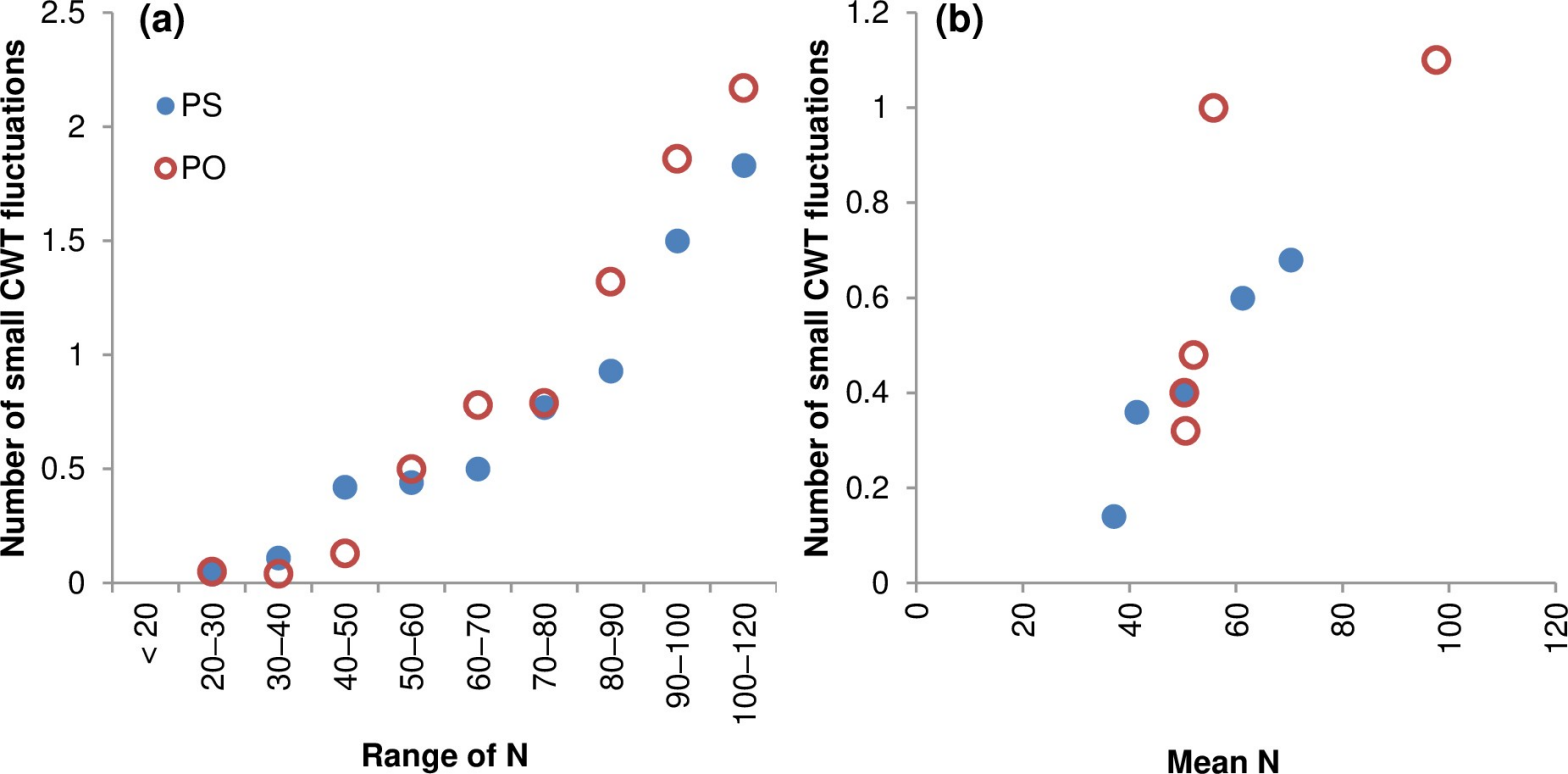
Relationships between cell number N and number of small CWT fluctuations per ring. (a) ratio of small CWT fluctuations’ number to the number of tree rings in different ranges of N; (b) mean values of N and ratio of small CWT fluctuations per ring for individual trees over 1965–2014. PS (filled markers), Scots pine (*Pinus sylvestris*); PO (empty markers), Siberian spruce (*Picea obovata*).

The numbers of the small CWT fluctuations in the tree rings of pine and spruce, averaged for groups of rings classified by total seasonal cell production, are presented in Fig 2A. It is easy to see that with an increase in the cell number (and subsequently tree-ring width), the number of observed small CWT fluctuations increases. According to our observations for very wide rings (>80 cells), 1–3 small CWT fluctuations are recorded in each ring. The numbers in Fig 2A are well described by linear approximation functions:

$$n_{pine}=0.0206\cdot \left(N-31\right),\;R=0.963,\;p<0.0001,$$

$$n_{spruce}=0.0241\cdot \left(N-31\right),\;R=0.967,\;p<0.0001.$$

It can be seen from the above equations that with cell production no more than 31 cells per growth season, small CWT fluctuations are unlikely (in the study area, one such fluctuation was recorded for each species in the range of N = 20–30 cells). This result approximately corresponds to the average production rate of 0.31–0.41 cells per day (1 cell per 2.4–3.3 days) and the maximum presence of 6–11 cells simultaneously in the zone of cell wall thickening. For pine rings, only 1–2 fluctuations per ring were observed even in the widest rings, but for spruce rings, 3 small CWT fluctuations per ring were sometimes observed for N>90.

The close relationship between the number of small CWT fluctuations and cell production is also supported by the data averaged for individual trees (Fig 2B). With an increase in the average growth rate, the number of small CWT fluctuations also increases (R = 0.96 and R = 0.75 for pine and spruce, respectively).

### Reflection of the small CWT fluctuations in wood density

Small CWT fluctuations should be distinguished from intra-annual density fluctuations (IADF), which are the subject of intense research on the conditions of the influence of various stress factors, such as intraseasonal droughts, on tree growth [10, 53, 54, 55, 56, 57]. First, IADFs are not observed in the study area, either in pine or in spruce xylem. Second, small CWT fluctuations have a lesser amplitude (up to 1 μm) compared to IADF and, as shown in Fig 1, may or may not be combined with corresponding fluctuations in D. Third, small CWT fluctuations occur more frequently in extremely wide rings formed under favorable conditions, i.e. they are not associated with severe stress as IADF. Partly because individual trees in the same year have different N, we were not able to statistically significantly identify the relationships of small CWT fluctuations with the climatic conditions of particular calendar years. Nevertheless, the dependence of the occurrence of small CWT fluctuations on N indicates that the reason for small CWT fluctuations is related to the cell production rate.

Any deviations in CWT, especially in latewood, are reflected in the wood density, since density is directly proportional to the ratio of the cell wall area to the total cell area [58]. However, these fluctuations may not be registered on the density profiles, since density is automatically averaged over several neighboring cells across the width of the optical probe for small latewood tracheids [59]. Such deviations in density profiles are more likely to be seen as noise.

### Possible reasons behind the small CWT fluctuations

Since small CWT fluctuations are synchronous within all measured radial files in the ring, they are characteristic of a specific ring in a particular tree, although they may not be observed in other trees or be asynchronous between trees for the same year. For wide tree rings where these fluctuations occur, a large number of cells can simultaneously be in the zone of cell wall thickening, thus having the same external conditions affecting the respective growth process. This result indicates that the observed deviations of CWT over 6–8 cells are unlikely to be driven by climatic fluctuations during secondary wall deposition. It is more probable that the climatic signal was somehow “picked up” by the cell during its time in the cambial zone, i.e., before transition to the cell expansion zone [12].

The process of tracheid differentiation is rather strictly regulated internally by the sequential activation of enzyme systems and genes, the end result of which is apoptosis [60, 61, 62, 63]. The two main processes of differentiation, cell expansion and cell wall thickening, are stretched in time; many cells are simultaneously located in the corresponding zones. Thus, only the transition of the cell from the cambial zone to maturation can be subject to a short-term influence of external conditions, even if tracheids leave the cambial zone in packets rather than one-by-one [64]. On the other hand, during the formation of IADF during a drought, a sharp decrease in the cell number in the cambial zone (i.e., in the cell production rate) is observed and then transmitted to zones of subsequent tracheid differentiation [10, 65]. This finding also supports the hypothesis of climatic signal registration in the cambial zone. However, since the small CWT fluctuations observed in the study area are not always accompanied by respective changes in D, the question of the transport mechanism of this signal remains open for further studies.

## Conclusion

Small fluctuations in latewood CWT observed in wide tree rings supports hypothesis that CWT tracheidogram register climatic variation not only during cell wall deposition, but also has input from conditions during previous stages of the tracheid differentiation, beginning from cambial activity. Thus, we offer a new fine-scaled tool in the range of methods for investigating the influence of internal and external factors on xylem structure formation: the analysis of small CWT fluctuations in tracheids of extremely wide conifer tree rings. We believe that automated measurements of conifer tracheidograms will expand the possibilities of such an analysis for proposing and testing new hypotheses about the regulation of xylem growth and differentiation.
